# Obstetric Outcomes and Delivery-Related Health Care Utilization and Costs Among Pregnant Women With Multiple Chronic Conditions

**DOI:** 10.5888/pcd15.170397

**Published:** 2018-02-08

**Authors:** Lindsay K. Admon, Tyler N. A. Winkelman, Michele Heisler, Vanessa K. Dalton

**Affiliations:** 1 National Clinician Scholars Program, University of Michigan, Ann Arbor, Michigan; 2 Institute for Healthcare Policy and Innovation, University of Michigan, Ann Arbor, Michigan; 3 VA Ann Arbor Healthcare System, Ann Arbor, Michigan; 4 Department of Obstetrics and Gynecology, University of Michigan, Ann Arbor, Michigan; 5 Program on Women’s Healthcare Effectiveness Research, University of Michigan, Ann Arbor, Michigan; 6 Departments of Internal Medicine and Pediatrics, Hennepin County Medical Center, Minneapolis, Minnesota; 7 Center for Patient and Provider Experience, Minneapolis Medical Research Foundation, Minneapolis, Minnesota; 8 Division of General Medicine, Department of Internal Medicine, University of Michigan, Ann Arbor, Michigan; 9 Department of Health Behavior and Health Education, University of Michigan School of Public Health, Ann Arbor, Michigan

## Abstract

Our objective was to measure obstetric outcomes and delivery-related health care utilization and costs among pregnant women with multiple chronic conditions. We used 2013–2014 data from the National Inpatient Sample to measure obstetric outcomes and delivery-related health care utilization and costs among women with no chronic conditions, 1 chronic condition, and multiple chronic conditions. Women with multiple chronic conditions were at significantly higher risk than women with 1 chronic condition or no chronic conditions across all outcomes measured. High-value strategies are needed to improve birth outcomes among vulnerable mothers and their infants.

## Objective

Complications from chronic conditions are a key driver of rising obstetric morbidity and mortality in the United States (1). The prevalence of chronic conditions, including multiple chronic conditions (MCCs), continues to rise among childbearing women (2,3). MCCs, defined as 2 or more chronic conditions, are associated with worse health outcomes, including death, and higher levels of health care utilization and costs among the non-pregnant adult population (4). Understanding these data influenced the development of high-value programs that improved health outcomes and lowered costs in high-risk, high-cost populations (5). Our objective was to measure obstetric outcomes and delivery-related health care utilization and costs among pregnant women with MCCs.

## Methods

We conducted a retrospective, cross-sectional analysis of 2013–2014 data from the National Inpatient Sample (6), a nationally representative sample of hospital discharges in the United States. Deliveries were identified by using previous methods (7), and data for chronic conditions were abstracted by using relevant codes from the *International Classification of Diseases, Ninth Revision, Clinical Modification* (ICD-9-CM) (8) (Box). We included 8 chronic conditions that are prevalent in the childbearing population and associated with obstetric morbidity and mortality: chronic respiratory disease, chronic hypertension, substance use disorders, pre-existing diabetes, chronic heart disease, chronic kidney disease, human immunodeficiency virus/acquired immune deficiency syndrome, and chronic liver disease (3,9).

***Box. International Classification of Diseases, Ninth Revision, Clinical Modification* Codes Used in Study**

**Table:** 

Condition	Codes
Chronic respiratory disease	491.x–496.x
Chronic hypertension	401.x–405.x, 642.0x, 642.1x, 642.2x, 642.7x
Substance use disorders	303.01, 303.02, 303.03, 304.x, 305.0x, 305.2x–305.9x, 648.3x
Pre-existing diabetes	249.x, 250.x, 648.0x
Chronic heart disease	412.x–414.x, 394.x-397.x, 424.x, 428.22, 428.23, 428.32, 428.33, 428.42, 428.43, 745.0x–747.4x, 648.5x
Chronic renal disease	581.x-583.x, 585.x, 587.x, 588.x, 646.2x
Chronic liver disease	571.x, 572.x
Human immunodeficiency virus/acquired immune deficiency syndrome	042.x, V08.x

Obstetric outcomes included preterm delivery, cesarean delivery, and severe maternal morbidity and mortality. Preterm delivery was measured by using ICD-9-CM code 644.21, and cesarean delivery was measured by using ICD-9-CM codes 740.x, 741.x, 742.x, 744.x, and 749.9. Severe maternal morbidity was measured by using standardized diagnosis and procedure codes outlined by the Centers for Disease Control and Prevention (10). Health care utilization measures included need for hospital transfer and length of stay. Delivery-associated hospital charges from the Healthcare Cost and Utilization Project’s cost-to-charge ratio files were used to calculate costs. Charge and cost estimates were adjusted for inflation to 2014 dollars.

We used multivariable logistic regression models to estimate obstetric outcomes. We generated estimates for hospital transfer, length of stay, and costs by using multivariable Poisson regression models, because these data were not normally distributed (Shapiro–Wilk test, *P* < .001 for each). All estimates were population averages generated with post-regression predictive margins and tabulated per 100 delivery hospitalizations. Models were adjusted for age, rural versus urban residence, primary insurance payer, median household income for the patient’s zip code, and hospital region. In sensitivity analyses of our cost estimates, we controlled for obstetric outcomes to isolate the independent association between MCCs and cost. We used complete case analysis rather than imputation, because less than 3% of observations had missing data. All analyses were conducted using STATA version 14.2 (StataCorp LLC). Our analysis of de-identified data was exempt from review by the study site’s institutional review board.

## Results

Our sample consisted of 1,508,413 unweighted delivery hospitalizations, representing 7,542,063 weighted delivery hospitalizations occurring nationally in 2013–2014. We identified MCCs in 12,567 unweighted delivery hospitalizations (weighted percentage, 0.83%; 95% confidence interval [CI], 0.80%–0.86%) and 1 chronic condition in 127,350 unweighted delivery hospitalizations (weighted percentage, 8.4%; 95% CI, 8.3%–8.6%). Women with MCCs were older than women with 1 chronic condition or no chronic conditions (30.2 y, 28.5 y, and 28.2 y, respectively) (Table 1). A higher proportion of women with MCCs (63.8%) had Medicaid as their primary payer compared with the proportion of women with 1 chronic condition (53.2%) or no chronic conditions (42.9%). 

**Table 1:** Characteristics of Patients With No Chronic Conditions, 1 Chronic Condition, and Multiple Chronic Conditions (Unweighted N = 1,508,413), National Inpatient Sample, 2013–2014^a^

Characteristic	No Chronic Conditions (n = 1,368,496)	1 Chronic Condition (n = 127,350)	Multiple (≥2) Chronic Conditions (n = 12,567)
**Mean age^b^, y**	28.2 (28.1–28.3)	28.5 (28.5–28.6)	30.2 (30.0–30.3)
**Payer**			
Medicaid	42.9 (42.1–43.7)	53.2 (52.3–54.1)	63.8 (62.5–65.1)
Private	51.3 (50.5–52.2)	42.4 (41.4–43.3)	32.2 (30.9–33.5)
Uninsured	5.8 (5.5–6.0)	4.4 (4.2–4.7)	4.0 (3.6–4.4)
**Bottom income quartile^c^**	27.1 (26.3–27.9)	33.4 (32.4–34.5)	40.4 (38.9–41.9)
**Rural residence**	14.1 (13.6–14.5)	14.6 (14.0–15.3)	13.7 (12.8–14.6)
**Hospital region**			
Northeast	15.9 (15.2–16.7)	17.6 (16.6–18.7)	17.2 (15.7–18.8)
Midwest	21.2 (20.4–22.0)	22.1 (21.0–23.1)	22.2 (20.7–23.9)
South	38.4 (37.4–39.5)	38.3 (37.0–39.6)	39.9 (38.0–41.9)
West	24.5 (23.6–25.4)	22.0 (21.0–23.1)	20.6 (19.3–22.0)

a All data presented as weighted percentage (95% confidence interval) unless otherwise noted.

b Weighted mean (95% confidence interval).

c Patients living in a zip code with a median household income in the bottom national income quartile.

Among pregnant women hospitalized for obstetric delivery, rates of preterm delivery, cesarean delivery, and severe maternal morbidity and mortality were significantly higher among women with MCCs than among women with no chronic condition or 1 chronic condition (Table 2). The rate of severe maternal morbidity and mortality among women with MCCs (6.4 per 100 delivery hospitalizations) was nearly 4 times higher than among women with no chronic conditions (1.7 per 100 delivery hospitalizations). Similarly, health care utilization and costs were highest among women with MCCs compared with those with no chronic condition or 1 chronic condition, even after controlling for obstetric outcomes and length of stay (Table 3). Women with 1 chronic condition were also at significantly higher risk than women with no chronic conditions across each outcome measured.

**Table 2:** Weighted National Estimates of Delivery-Related Outcomes Among Patients With No Chronic Conditions, 1 Chronic Condition, and Multiple Chronic Conditions, National Inpatient Sample, 2013–2014^a^

Outcome	No Chronic Conditions	1 Chronic Condition	Multiple (≥2) Chronic Conditions
**Health outcomes**			
Preterm delivery (<37 weeks)	5.7 (5.6–5.7)	9.7 (9.5–10.0)	15.1 (14.4–15.9)
Cesarean delivery	31.9 (31.7–32.2)	41.2 (40.8–41.6)	53.3 (52.3–54.2)
Severe maternal morbidity and mortality	1.7 (1.7–1.7)	3.0 (2.8–3.1)	6.4 (5.9–6.8)
**Health care use**			
Hospital transfer	1.1 (0.9–1.2)	2.0 (1.8–2.3)	3.5 (3.1–4.0)
Length of stay, in days^b^	2.6 (2.6–2.6)	3.1 (3.1–3.2)	4.3 (4.2–4.3)
**Health care expenditures, **$^c^			
Mean charges per delivery hospitalization	16,000 (16,000–17,000)	20,000 (19,000–20,000)	28,000 (27,000–29,000)
Mean cost per delivery hospitalization	4,500 (4,400–4,500)	5,600 (5,500–5,600)	7,700 (7,500–8,000)

a All data presented as rate per 100 delivery hospitalizations (95% confidence interval) unless otherwise noted. Adjusted for age, rural vs urban residence, payer, national income quartile for zip code of residence, and hospital region.

b Weighted mean (95% confidence interval).

c Inflation-adjusted to 2014 US dollars.

**Table 3:** Weighted National Estimates of Mean Cost (95% CI)^a^ per Delivery Hospitalization Among Patients With No Chronic Condition, 1 Chronic Condition, and Multiple Chronic Conditions, National Inpatient Sample, 2013–2014

Model Adjustment^b^	No Chronic Conditions	1 Chronic Condition	Multiple (≥2) Chronic Conditions
Severe maternal morbidity and mortality	4,500 (4,400–4,500)	5,500 (5,400–5,600)	7,300 (7,100–7,500)
Preterm delivery	4,500 (4,400–4,500)	5,500 (5,400–5,600)	7,400 (7,200–7,600)
Cesarean delivery	4,500 (4,400–4,600)	5,300 (5,200–5,400)	6,900 (6,700–7,100)
Length of stay	4,500 (4,400–4,500)	5,500 (5,400–5,600)	7,400 (7,200–7,600)
All outcomes	4,500 (4,500–4,600)	5,200 (5,100–5,200)	6,200 (6,000–6,400)

Abbreviation: CI, confidence interval.

a Inflation-adjusted to 2014 US dollars.

b In addition to the following demographic covariates: age, rural vs urban residence, payer, national income quartile for zip code of residence, and hospital region.

## Discussion

In this nationally representative sample of delivery hospitalizations from 2013–2014, women with MCCs had worse health outcomes, higher levels of health care utilization, and greater hospital costs associated with obstetric delivery compared with women with no chronic conditions or 1 chronic condition. In particular, the rate of severe maternal morbidity and mortality was 276% higher among women with MCCs than among women with no chronic conditions. Hospital costs were highest among patients with MCCs compared with those with no chronic condition or 1 chronic condition — even after even after controlling for obstetric outcomes and length of stay. It is plausible that higher costs may have resulted from greater use of consultative or social services, data that were not captured by our methods.

A limitation of our study design is that clinicians were likely to document ICD-9-CM codes only for conditions that were addressed during a delivery hospitalization. As such, our point estimates of MCC prevalence are likely to be conservative. For example, some women with MCCs may have been misclassified as having 1 chronic condition or no chronic conditions. Total costs associated with MCCs are also likely to be larger than our estimates. For example, women with MCCs had higher rates of preterm delivery, and considerable neonatal costs are associated with preterm birth (11).

Our findings show that pregnant women with MCCs are a high-risk, high-cost population. The strong association between MCCs and worse delivery-related health outcomes found in our study suggests that MCCs may contribute to the high levels of maternal morbidity and mortality in the United States. MCCs also contribute significantly to the cost of delivery hospitalizations. Delineating the highest-risk and highest-cost combinations of conditions may provide crucial data for the development of high-value strategies to improve birth outcomes among these vulnerable mothers and their infants.
